# Effectiveness of Specific Professional Training in Male Elite Adolescent Team Handball Players

**DOI:** 10.3390/sports13060193

**Published:** 2025-06-19

**Authors:** Wagner Herbert, Radic Vanja, Hinz Matthias

**Affiliations:** 1 Department of Sport and Exercise Science, University of Salzburg, 5020 Salzburg, Austria; 2 Department of Sport Science, Otto von Guericke University of Magdeburg, 39106 Magdeburg, Germany; matthiashinz87@yahoo.com; 3 Team Handball Club Dessau-Roßlauer HV, 06844 Dessau-Roßlau, Germany; radicvanja@hotmail.de

**Keywords:** training, testing, physical training, team handball match, oxygen uptake, blood lactate

## Abstract

Professional training in elite team handball academies is key to developing top players, yet its direct impact on physical performance remains unclear. This study aimed to (1) provide professional training to elite adolescent players and (2) assess performance improvements using a team handball-specific test. Thirty elite male players (six goalkeepers, 24 field players) participated in an 11-week program, with nine under-19 (17.2 ± 1.3 years) and nine under-17 (15.6 ± 0.9 years) field players completing at least 80% of sessions. All underwent pre- and post-testing using the game-based performance test. A two-way ANOVA analyzed differences between tests and age groups as well as playing positions. Significant improvements (*p* < 0.05) were found in defense and offense time and body weight for both groups. Under-17 players also showed a significant increase in peak oxygen uptake (+9%), ball velocity (+7%), and jump height (+20%). Agility in defense and offense improved in under-19 (+3%) and under-17 (+6%) players, aligning with training goals. Positional differences were observed between backcourt players and wings (*p* < 0.01) in the ball velocity, while all players showed improvements in both defense and offense performance. We suggest that professional and targeted specific training at this age has a significant impact on the long-term development of adolescent team handball players and is the basis for a professional handball career.

## 1. Introduction

Team handball is an Olympic team sport played on an indoor court measuring 40 × 20 m^2^. Characterized by fast-paced, intermittent defensive and offensive actions, the game’s objective is to score more goals than the opposing team. A standard match consists of two 30-min halves, during which players cover distances between 3900 and 4700 m. Among elite male team handball players, relative workloads typically range from 70–80% of maximal oxygen uptake (VO2max) throughout a match, with workload reductions commonly seen in the second half. Each field player (six field players and one goalkeeper per team) undergoes numerous intensity shifts and technical demands, including direction changes (25–35 per match), jumps (10–20), throws on goal (5–10), passes (40–60), and both light (45–55) and hard (10–15) body tackles [1,2]. These high-intensity actions alternate with periods of low-intensity movement, such as walking and standing, which comprise 70–75% of total playing time. Notably, elite players spend only 1–2% of total playing time sprinting [1,3,4]. Due to their position-specific roles, backcourt players, wings, and pivots differ in total playing time and total distance covered (with wings playing longer), body weight (pivots being heavier), as well as sprinting and throwing performance (wings performing more sprints, while backcourt players achieve higher ball velocities) [4,5,6,7], with these differences generally more pronounced in adult players compared to adolescents [8,9,10,11]. However, a switch between playing positions during adolescence is not uncommon, and some of the world’s best wings or pivots previously played as backcourt players (backs) in their younger years. In practice, elite training in adolescent team handball tends to be less position-specific compared to the more specialized training seen at the adult elite level.

In male team handball, professional training for adolescent players is crucial for developing top-elite athletes. Such training typically involves 7–8 sessions per week, including general physical and specific team handball training, along with participation in 1–2 national or international top-level matches. Despite the rigorous frequency and intensity of this training regimen, its effectiveness is often assessed through coach observations during training and matches, as well as through general physical performance tests [10,11,12,13,14,15,16,17,18,19]. However, the aim of professional training in adolescent team handball is to enhance specific physical performance. Therefore, accurately measuring this performance is essential to assess potential improvements. A test to determine this specific physical performance is the team handball game-based performance test (GBPT), which has been validated in previous studies [20,21]. In elite adolescent team handball players, Wagner, Hinz [22] found a correlation between increasing age and specific team handball performance. Furthermore, the GBPT was used to assess the adaptation of specific performance to specific physical training in a one-year training study involving elite adult male team handball players [23]. Evaluating the effects of professional training in adolescent team handball within a controlled study can provide valuable insights into optimal training planning for this age group. In this context, it is also relevant to investigate whether players of different age groups and playing positions (backs, wings, and pivots) respond differently to the training stimulus.

Consequently, the aims of the study were (1) to provide professional training to elite adolescent team handball players, and (2) to measure their performance improvement utilizing the GBPT. We hypothesized that the professional training regimen would lead to measurable improvements in specific physical performance among elite adolescent team handball players, with significant differences in improvement between age groups (under-19 and under-17) and playing positions (backs, wings, and pivots).

## 2. Materials and Methods

### 2.1. Participants

**Figure 1:**
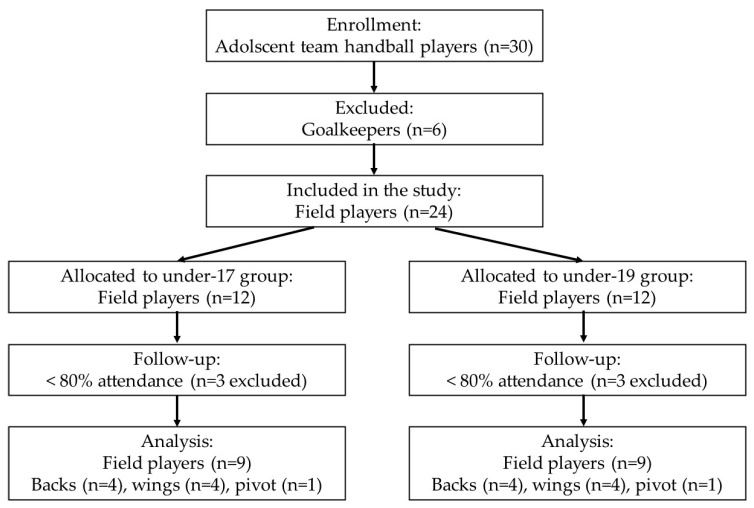
Consort flow allocation of the study.

Thirty adolescent male team handball players, consisting of six goalkeepers and 24 field players, were involved in this study. Only field players took part in the GBPT. Inclusion criteria for field players required attendance at a minimum of 80% of all training sessions during the study period. Consequently, the study included (Figure 1) nine under-19 players (age: 17.2 ± 1.3 years, body weight: 77.8 ± 6.0 kg, body height: 1.82 ± 0.04 m) and nine under-17 players (age: 15.6 ± 0.9 years, body weight: 71.5 ± 14.5 kg, body height: 1.82 ± 0.06 m).

All participants included in the study were deemed physically healthy and reported no injuries that would prevent them from participating in team handball training or matches during the study period. The Ethics Committee of the University of Salzburg approved the research protocol (reference number: GZ-44-2015) in accordance with the Declaration of Helsinki. Prior to participation, all participants signed informed consent. For players under 18 years of age, their legal guardians reviewed and signed the informed consent.

### 2.2. Study Design

This study constituted a quasi-experimental study spanning an eleven-week training period, encompassing both the pre-season preparation phase and the initial three games of the highest under-19 and under-17 German Team Handball League’s official season. The training regimen comprised 2–3 physical sessions and 4–5 specific team handball training sessions, amounting to a total of 7–8 sessions per week, along with 1–2 matches weekly. To assess performance improvement, all participants underwent the GBPT twice: once prior to the training period (pre-testing) and once after its completion (post-testing).

### 2.3. Team Handball Game-Based Performance Test (GBPT)

Before testing, participants completed an eight-minute general warm-up (jogging) and a fifteen-minute handball-specific warm-up involving passing, throwing, directional changes, and light contact. The GBPT began with one familiarization heat at ~70% effort, followed by eight test heats featuring handball-specific offensive and defensive actions, transitions, and active recovery phases [21].

**Figure 2:**
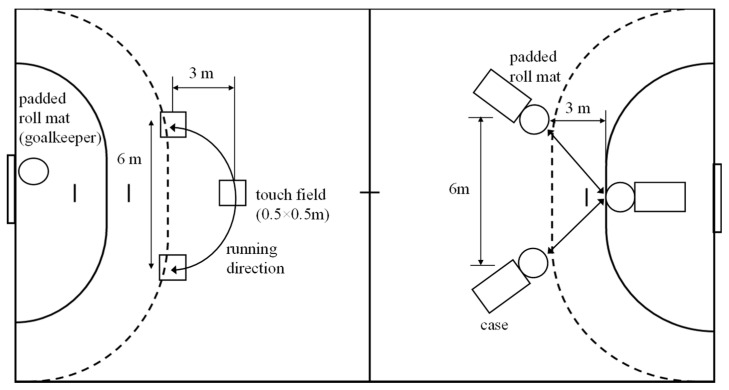
Schematic figure of the playing court in the team handball game-based performance test. Adapted from a previously published study [24].

In defense, participants sprinted three times from the 6 m to 9 m line, simulating tackles by touching padded roll mats (Figure 2) with both hands. In offense, they sprinted from the 12 m to 9 m line three times, touching 0.5 × 0.5 m floor markers with one foot while catching and passing the ball (Figure 2). In heats #2, #3, #4, #6, and #8, participants completed offensive actions with a one-legged jump shot, aiming for maximum throwing speed into the bottom corner of the goal. In heats #3 and #6, participants performed fast breaks by sprinting from defense to offense, receiving the ball at 12 m, and finishing with a jump shot at 9 m. In heats #4 and #6, they executed fast retreats from offense to defense immediately after the jump shot. A detailed description of the different movements in all heats has been published in a previous validation study of the GBPT [21].

Defense time (first to last contact on padded mats), offense time (contacts on floor markers), 10 m-fast break time (from 9 m defense line to midline), and 20 m-fast break time (midline to offensive floor markers) were measured using three manual stopwatches (Hanhart Stratos 2, Hanhart GmbH, Gütenbach, Germany) [20,25]. Fast retreat time was excluded due to variable acceleration after jump shots. Break durations, 20 s (between offense and defense), 15 s (between two actions), and 40 s (between heats), were controlled using Multi-Timer-Ultimate 3.1 software, with an audible countdown in the final 3 s. Jump shots were recorded at 200 fps (JVC-GC-PX100BE, Yokohama, Japan), and analyzed with Tracker 4.59 software (Douglas Brown, Aptos, CA, USA) to calculate ball velocity and jump height. Calibration used a 4-point reference from the goal (2 × 3 m). Flight time was defined as the interval between take-off and landing foot contact. Ball velocity was calculated from 2D motion over 15 frames, and jump height from flight time using gravitational acceleration. The mean values of the best three attempts were used for analysis [20].

$$h_{jump}=\frac{g}{8}\times t_{flight}^{2}$$

During the GBPT, heart rate and oxygen uptake were measured using a heart rate belt (Suunto T6d, Suunto, Vantaa, Finland) and a portable breath-by-breath gas analysis system (K5, Cosmed, Rome, Italy). Peak heart rate (HRpeak) and peak oxygen uptake (VO2peak) were calculated across all heats. For VO2peak, only values surrounded by two consecutive measurements above 90% of the peak were included to ensure accuracy [25].

### 2.4. Training Program

**Figure 3:**
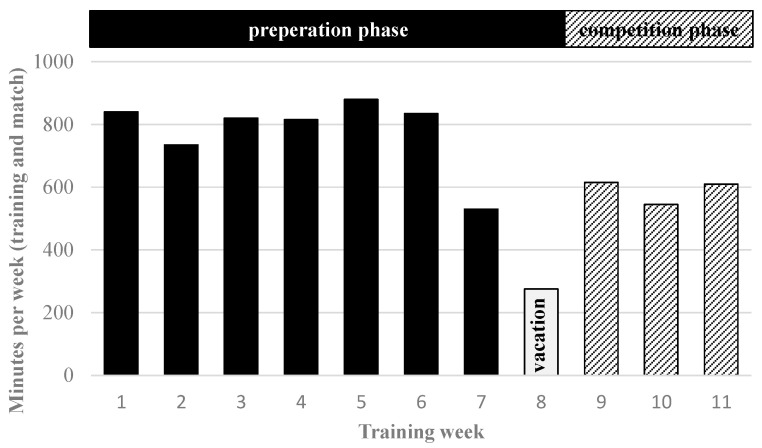
Total minutes per week (including training and matches) during the eleven-week training program, encompassing the preparation and competition phases as well as a vacation week, for the under-17 players.

**Figure 4:**
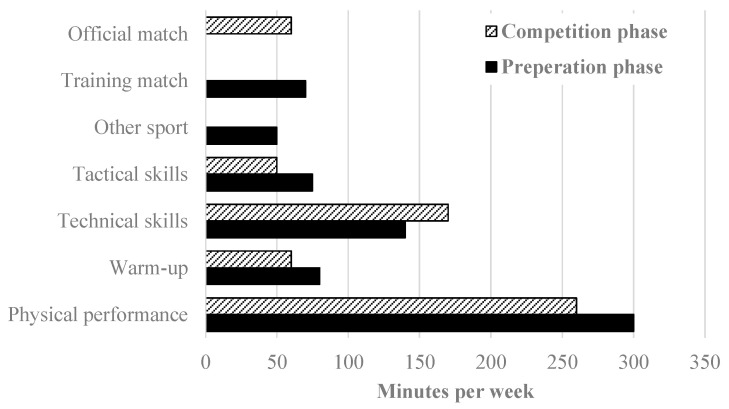
Mean minutes per week for the seven training focuses, separated by competition and preparation phases, in the under-17 players.

As outlined in the study design, the training program included an eight-week pre-season preparation phase and a three-week competition phase (Figure 3), which encompassed the first three games of the official season in Germany’s highest-team handball league. To structure the training effectively, seven distinct training focuses were established:

Warm-up: The warm-up phase was designed as optimal preparation for subsequent training activities, incorporating several components: individual run-in exercises, running ABC drills (e.g., heel kicks, knee lifts, step jumps, hop runs, and side-steps), ball-handling drills (e.g., bouncing, juggling, and passing), coordination exercises using ladders, rings, and benches, preparatory exercises such as complex passing drills, and/or warm-up games (e.g., small-sided games). In the competition phase, the average weekly warm-up time was 60 min, whereas, in the preparation phase, it was 80 min (Figure 4), due to the higher training volume during the preparation phase (Figure 3).

Other sport: To introduce variation into the training process, other sports such as soccer, basketball, and volleyball along with various athletic disciplines like sprinting, long jump, shot put, javelin, and swimming, were incorporated into the preparation phase.

Physical performance: Physical performance training plays a crucial role in optimizing preparation for high-performance training in elite adult team handball, particularly at this developmental stage [17,22,26]. Therefore, it was a primary focus during training sessions. This training encompassed various components, including low-intensity endurance (high volume), high-intensity interval training (both general and team handball-specific, featuring different interval lengths), sprint training, repeated sprint ability, agility training, as well as sensomotoric, mobility, and flexibility training, alongside strength and power training. Within strength and power training, particular emphasis was placed on barbell exercises such as back squats, front squats, overhead squats, split squats, deadlifts, bench presses, cleans, and power cleans. Additionally, to develop jumping and throwing power, exercises like box jumps, medicine ball drills, and elastic band exercises were incorporated. Furthermore, dumbbells and stationary strength training equipment available in the academy’s gym were utilized to enhance overall strength and power. In the competition phase, the average weekly time for physical performance training was 260 min, compared to 300 min in the preparation phase (Figure 4), due to the higher training volume during the preparation phase (Figure 3). During the autumn break (vacation), the players had a week of home leave (training week #8), during which they were required to complete a home training program. This program included a 45-min warm-up and 200 min of physical performance training per week (Figure 4).

Technical skills: Team handball technical skills included basic attacking skills such as throwing, passing, faking, and feinting; complex attacking cooperations like crossing, cutting in, direct or indirect blocking, pushing, and rebounding; complex defending cooperations including closed, open, deep, or pressing defense; transitions such as fast transitions, fast breaks, fast throw-offs (defense to offense), and quick retreats (offense to defense); as well as goalkeeper training that involved drills with field players. During the preparation phase, the average training time for technical skills was 140 min per week. In the competition phase, this increased to 170 min per week due to a greater emphasis on team handball-specific training.

Tactical skills: Team handball tactical skills training included one-on-one drills and small group games (two-on-two, three-on-three, and four-on-four), attacking tactics involving all field players (six-on-six), as well as fast transition tactics. During the preparation phase, the average training time for tactical skills was 75 min per week; however, during the competition phase, it was reduced to 50 min per week. The increase in volume (+25 min) during the preparation phase was aimed at developing attacking tactics. For optimal training, team handball-specific sessions often combined warm-up, sensomotoric, mobility, and flexibility training with technical and tactical skills training. These sessions were structured with an optimal sequence of exercises, ensuring that individual drills built on one another.

The technical and tactical skills training used in the present study is typical for this age group in elite adolescent team handball. It was based on the training concept of the German Handball Federation and complemented by exercise variations from experienced coaches with the highest coaching license (Master Coach of the European Handball Federation). A detailed description of the technical and tactical training would be too extensive; however, some of these skills are described in detail (including video clips) in a book on modern methods for team handball-specific training [27].

Training match: Training matches during the preparation phase included standard training matches (50 to 60 min against rival teams), participation in national tournaments (2–3 matches over two days against other national teams), and, as a highlight, the German International Youth Championship in Düsseldorf, an international handball tournament featuring the top European teams in this age category. The average training match duration per week during the preparation phase was 70 min.

Official match: The official matches consisted of the first three games of the official season in the highest German Team Handball League, with each game having a duration of 2 × 30 min for under-19 players and 2 × 25 min for under-17 players.

External factors: All participants in the present study were athletes from a local elite team handball academy, where they followed a standardized daily schedule. This included structured training sessions, school attendance, study periods, rest, and sleep routines, with bedtimes adjusted according to age. Nutrition was consistently provided through the academy’s canteen, including breakfast, mid-morning snack, lunch, afternoon snack, and dinner. No dietary supplements were consumed during the study period.

### 2.5. Statistical Analysis

All statistical analyses were conducted using SPSS version 29.0 (SPSS Inc., Armonk, NY, USA). Means and standard deviations were calculated for descriptive statistics. To assess differences between pre- and post-test results in the under-17 and under-19 players, a two-way repeated-measures ANOVA was employed, with “time” and “team” as well as “time” and “position” as the main factors. A Bonferroni post hoc test was used to identify significant differences between the under-17 and under-19 players as well as the playing positions, and effect size (η^2^) and power (1 − ß) were calculated. For all statistical analyses, significance was set at *p* < 0.05, and the effect size was defined as small (0.01 ≤ η^2^ < 0.06), medium (0.06 ≤ η^2^ < 0.14), and large (η^2^ ≥ 0.14) [28].

## 3. Results

Mean values ± standard deviation and 95% confidence intervals for the under-19 and under-17 players (pre- and post-test) in the team handball game-based performance test are depicted in Table 1.

Significant differences were found between the under-19 and under-17 players in the 10 m-fast break time (*p* < 0.01) and defense time (*p* < 0.001) for the factor “team”; significant differences were also observed between pre- and post-test in defense time (*p* < 0.001), offense time (*p* < 0.001), and jump height (*p* < 0.01) for the factor “time,” as well as an interaction effect between team and time (*p* < 0.05) for defense time. In addition to these significant differences in team and time with a large effect size and the interaction effect of team and time with a medium effect size, a medium effect size was also found in jump height and body mass for the factor “team,” in ball velocity for the factor “time,” and in VO2peak, 20 m fast-break time, and offense time for the interaction effect of team and time (Table 1).

**Figure 5:**
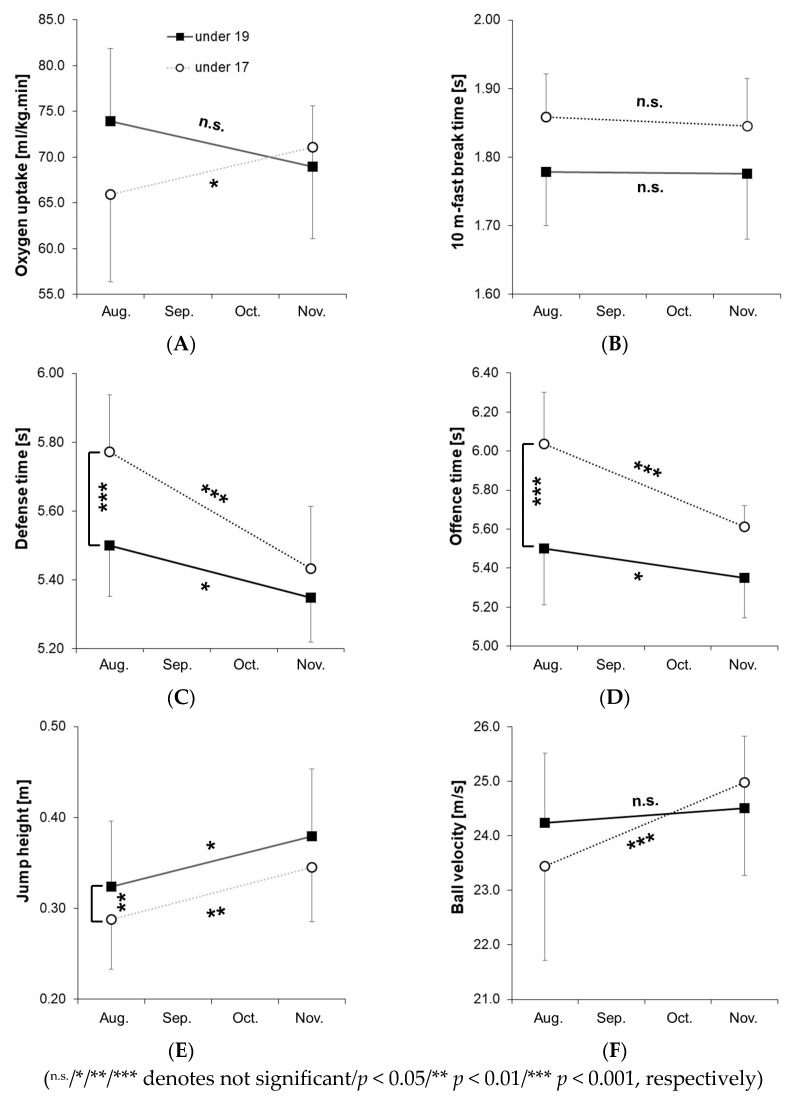
Mean values ± standard deviation for under-19 and under-17 players (pre-test in August, post-test in November) in the team handball game-based performance test for peak oxygen uptake (**A**), 10 m fast-break time (**B**), defense time (**C**), offense time (**D**), jump height (**E**), and ball velocity (**F**) in the jump shot.

For a detailed visualization of the results, mean values ± standard deviations for VO2peak, 10 m-fast break time, defense and offense time, jump height, and ball velocity in the jump shot are shown in Figure 5A–F. Post hoc test results are indicated with asterisks (* *p* < 0.05, ** *p* < 0.01, *** *p* < 0.001) in the figures. Among under-19 players, a decrease of 6.7% in VO2peak, a decrease of 0.2% in the 10 m-fast break time, a decrease of 2.7% in defense and offense time, along with an increase of 17.0% in jump height, and 1.1% in ball velocity, were observed. For under-17 players, we observed an increase of 8.8% in VO2peak, a decrease of 0.7% in 10 m-fast break time, a decrease of 5.9% in defense time, a decrease of 7.0% in offense time, along with an increase of 19.9% in jump height and 6.5% in ball velocity.

**Figure 6:**
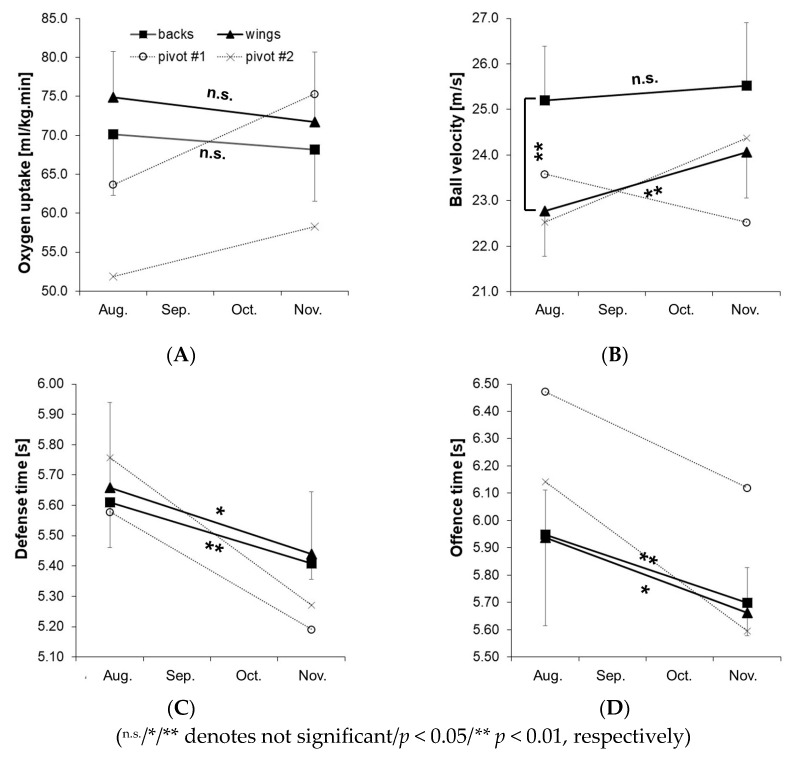
Mean values ± standard deviation for backs and pivots (pre-test in August, post-test in November), as well as the values of the under-19 pivot (pivot #1) and under-17 pivot (pivot #2) in the team handball game-based performance test for peak oxygen uptake (**A**), ball velocity in the jump shot (**B**), defense time (**C**), and offense time (**D**).

Due to the requirement of a minimum of 80% attendance in all training sessions, two backs and one pivot from each age group were excluded from the final analysis. As a result, data from eight backs, eight wings, and only two pivots were available to assess positional differences. Consequently, the two-way ANOVA with the factors “time” and “position” was conducted only for backs and wings. Figure 6A–D presents the mean ± standard deviation values for backs and wings in VO2peak, defense time, offense time, and ball velocity during the jump shot. For descriptive purposes, the individual values of the two pivots were also included in Figure 6A–D to illustrate potential training effects in this position.

Significant differences between backs and wings were observed in ball velocity during the jump shot (Figure 6B; F = 10.07, *p* < 0.01, η^2^ = 0.27, 1-ß = 0.86), as well as in defense time (Figure 6C; F = 11.95, *p* < 0.01, η^2^ = 0.30, 1-ß = 0.92) and offense time (Figure 6D; F = 13.28, *p* < 0.01, η^2^ = 0.32, 1-ß = 0.94) for the factor “time.” Post hoc tests revealed significant improvements in both wings (*p* < 0.05) and backs (*p* < 0.01) in defense and offense time. No significant effects for the factors “position” or “time” were found for VO2peak (Figure 6A), 10 m and 20 m fast break time, or jump height during the jump shot. Additionally, there was no significant interaction effect between “time×position” in the two-way ANOVA. Descriptive results for the two pivots showed similar trends to backs and wings in defense and offense time but differed in VO2peak (both pivots improved) and in ball velocity (Figure 5A), where the under-17 pivot improved while the under-19 pivot showed a decrease (Figure 5B).

## 4. Discussion

The aims of the study were to (1) provide professional training to elite adolescent team handball players and (2) measure their performance improvement using the GBPT. As expected, we found significant performance increases in both under-19 and under-17 players in defense time, offense time, and jump height during the jump shot (Table 1). Training for these age groups is designed to incorporate numerous fast movements specific to team handball scenarios (technical and tactical skills) as well as physical conditioning. At this developmental stage, enhancing offensive and defensive skills (including acceleration, deceleration, directional changes, and tackling) and jump shot performance is crucial for advancing to elite male team handball levels [22]. Interestingly, this improvement in performance was not observed in straight sprinting movements (10 m and 20 m fast-break time). However, straight sprinting is not strongly related to offense and defense in team handball [25] and has less impact on handball-specific performance [20,22,29]. Consequently, agility, speed, and power training in team handball should be tailored to enhance specific defensive, offensive, and jumping skills [26]. Notably, under-17 players showed greater improvements in defense and offense than under-19 players (Figure 5C,D). A performance gap in defense (0.31 s) and offense (0.25 s) was observed between the under-19 and under-17 players, suggesting that younger athletes may have a greater potential for performance gains.

An increase in ball velocity was observed only in under-17 players, not in under-19 players, despite the fact that post-test ball velocity was higher in under-17 players than in under-19 players (Figure 5F). This difference may be due to the smaller ball size and lighter weight used in the under-17 age group. According to the International Handball Federation (IHF) rules, ball size 3 (volume: 55–60 cm; weight: 425–475 g) is designated for adult male players and adolescent players over 16 years, while ball size 2 (volume: 54–56 cm; weight: 325–375 g) is intended for adult female players and male players aged 12 to 16. Following these guidelines, the German Handball Federation specifies ball size 3 for under-19 and ball size 2 for under-17 players. We propose that the reduced ball volume and weight for under-17 players contributed to the significant increase (*p* < 0.001) of 6.5% in ball velocity, as observed through targeted training (technical, tactical, and official matches) during the preparation and competition phases of the study.

When measuring VO2peak during the GBPT, no significant difference was found in under-19 players, but a significant increase (*p* < 0.05) was observed in under-17 players from pre-test to post-test. This increase in VO2peak among under-17 players may be attributed to high-intensity training in the preparation phase [23,26], which included (1) physical performance training, such as sprints, accelerations, decelerations, and changes of direction; (2) technical skills training, including throwing, passing, feinting, complex attacking and defending maneuvers, and fast transitions (fast breaks, throw-offs, and quick retreats); and (3) tactical skills training, including one-on-one drills, small group games, and fast transition tactics. In the elite handball academy of the present study, the long-term training approach prioritizes individual development for under-17 players (e.g., in official matches, the entire team was rotated in blocks to allow equal playing time). In contrast, for under-19 players, the focus shifts to team performance aimed at winning games and championships. We propose that this competitive focus for under-19 players may reduce high-intensity events, as it emphasizes six-on-six team skills over individualized high-intensity activities, although training intensity was not directly measured.

When analyzing positional differences, both backs (*p* < 0.01) and wings (*p* < 0.05), as well as both pivots, showed improved performance in defense and offense time. This suggests that the physical, technical, and tactical training applied in the present study effectively enhanced both defensive and offensive skills across all playing positions and age groups [10,11]. Notably, the improvement in defense time was particularly pronounced in the pivots, who outperformed their age-group peers in the post-test. Given the teams’ preferred 6:0 defensive tactic, where pivots typically play in the central block requiring quick lateral movements, we attribute these gains to targeted defensive elements in the technical and tactical training [27]. As expected, backs demonstrated the highest ball velocity in the jump shot [8,9,17]. However, wings (*p* < 0.01) and the under-17 pivot showed substantial improvement, approaching the performance level of the backs from pre- to post-test. No significant changes in VO2peak were observed for backs and wings, but both pivots demonstrated improvements. These findings suggest that the specific training program not only enhances overall performance but also helps address positional or individual deficits, such as VO2peak in pivots or ball velocity in wings and under-17 players. To our knowledge, this study is the first to measure the direct impact of elite team handball training on specific physical performance, which is crucial for planning effective training in elite handball academies. However, the study also has limitations. While we documented the training as accurately as possible, we did not measure objective training intensity. For future studies, local position measurement systems and physiological metrics such as heart rate or blood lactate could provide more accurate intensity data. However, measuring load parameters such as metabolic power through local positioning systems has limited accuracy in team handball [30] and physiological metrics (e.g., heart rate, blood lactate) vary widely among individuals [20,29], making them impractical for this setting. Although training workload can also be assessed using perceived exertion, we chose not to include this measure. Instead, the team handball academy employed a long-established and proven monitoring system. Each week, the head coach, assistant coach, goalkeeper coach, physical coach, and physiotherapist collectively discussed workload, performance status, and stress levels for each player to prevent overload. This system was also applied in the present study and led to the exclusion of two players due to a heightened risk of overload. Additionally, four other players were excluded due to injuries and illness. Another limitation of the study was the small sample size, which prevented statistical evaluation of training effects for pivots. Nevertheless, the value of assessing training outcomes in elite adolescent team handball players outweighs this limitation.

## 5. Conclusions and Practical Implications

We conclude that the training regimen in the present study, which comprised 2–3 physical training sessions and 4–5 specific team handball sessions per week, totaling 7–8 sessions per week, along with 1–2 weekly matches, is an effective approach for enhancing the specific performance of male under-19 and under-17 players. We suggest that structured, targeted training at this developmental stage has a significant impact on the long-term development of adolescent team handball players and lays the foundation for a professional handball career. For example, the under-17 pivot showed the lowest VO2peak and was among the weakest performers across most variables in the pre-test. After receiving individualized feedback comparing his results to the mean values of the other under-17 players, he not only improved his performance by the post-test but also significantly changed his training attitude. As a result, he was later selected for the national team and won a medal at the European Junior Championships. For coaches working with adolescent team handball players, we recommend implementing a well-balanced training program that integrates physical performance, technical, and tactical skills, like the training program used in the present study. To evaluate training outcomes, we advise using tests that assess team handball-specific physical performance. If the GBPT is too time- or cost-intensive, we suggest using its defense and offense components to measure defense time, offense time (requiring only a manual stopwatch), jump height, and ball velocity in the jump shot (using the free Tracker software and a video camera). For assessing specific aerobic performance, we recommend the YoYo Intermittent Recovery Test, which has shown a strong correlation (r = 0.56) with VO2peak as measured by the GBPT in elite female handball players [31].

The training program used in the present study was tailored for elite adolescent team handball players; however, the majority of adolescent players worldwide participate at a non-elite level. For these athletes, social interaction through team handball and personal fitness are often more relevant, both of which have significant implications for public health. Adapting the general structure of this training program by balancing physical conditioning with technical and tactical skills, to match the performance level of non-elite players may also improve their anaerobic and aerobic performance. Enhancing physical performance during adolescence contributes to better fitness in adulthood, even if players discontinue team handball later. Therefore, the balanced training approach presented here has the potential to help preserve and improve anaerobic performance in adolescent players more broadly, contributing to long-term health benefits and reducing performance disparities across European countries [32,33].

## Figures and Tables

**Table 1:** Mean ± standard deviation (SD) and 95% confidence intervals (CI) in the under 19 and under 17 players (pre- and post-test) in the team handball game-based performance test in the peak oxygen uptake (VO2peak), peak heart rate (HRpeak), 10 and 20 m-fast break time, defense and offense time, jump height and ball velocity in the jump shot, as well as body mass and body height. F-values, *p*-values, and effect size for the factors team, time, and team × time in the two-way repeated-measures ANOVA.

	Under-19 Player		Under-17 Players				
	Pre-Test Mean ± SD (95% CI)	Post-Test Mean ± SD (95% CI)	Pre-Test Mean ± SD (95% CI)	Post-Test mean ± SD (95% CI)	Team F-, *p*-Value Effect Size	Time F-, *p*-Value Effect Size	Team × Time F-, *p*-Value Effect Size
VO_2_peak (mL/kg.min)	73.9 ± 7.9 (68.1–79.8)	69.0 ± 6.6 (63.1–74.8)	65.9 ± 9.6 (60.1–71.8)	71.1 ± 10.0 (65.2–77.0)	1.03, 0.32 0.03	0.01, 0.98 0.00	3.13, 0.09 0.09
HRpeak (bpm)	192 ± 5 (189–196)	192 ± 9 (187–197)	193 ± 6 (188–198)	193 ± 7 (188–197)	0.21, 0.65 0.01	0.00, 0.98 0.00	0.05, 0.83 0.00
10 m-fast break time (s)	1.78 ± 0.08 (1.73–1.83)	1.78 ± 0.10 (1.72–1.83)	1.86 ± 0.06 (1.81–1.91)	1.85 ± 0.07 (1.79–1.90)	7.75, <0.01 ** 0.19	0.21, 0.65 0.01	0.10, 0.75 0.00
20 m-fast break time (s)	1.75 ± 0.10 (1.68–1.93)	1.79 ± 0.12 (1.71–1.86)	1.86 ± 0.10 (1.78–1.94)	1.72 ± 0.14 (1.66–1.81)	0.68, 0.41 0.02	1.26, 0.27 0.04	4.03, 0.05 0.11
Defense time (s)	5.50 ± 0.15 (5.40–5.60)	5.35 ± 0.13 (5.25–5.46)	5.77 ± 0.16 (5.67–5.88)	5.43 ± 0.18 (5.30–5.51)	10.41, <0.001 *** 0.25	25.31, <0.001 *** 0.44	4.49, 0.04 * 0.12
Offence time (s)	5.92 ± 0.29 (5.76–6.07)	5.76 ± 0.20 (5.60–5.91)	6.04 ± 0.27 (5.88–6.19)	5.61 ± 0.11 (5.45–5.76)	0.03, 0.87 0.00	15.37, <0.001 *** 0.32	3.17, 0.08 0.09
Jump height (m)	0.32 ± 0.07 (0.28–0.37)	0.38 ± 0.07 (0.34–0.42)	0.29 ± 0.05 (0.24–0.33)	0.34 ± 0.06 (0.31–0.40)	2.14, 0.15 0.06	7.97, <0.01 ** 0.20	0.07, 0.80 0.00
Ball velocity (m/s)	24.2 ± 1.3 (23.2–25.2)	24.5 ± 1.3 (23.5–25.5)	23.4 ± 1.7 (22.4–24.5)	25.0 ± 1.7 (23.8–25.8)	0.25, 0.62 0.01	2.79, 0.10 0.08	1.22, 0.28 0.04
Body mass (kg)	78 ± 6 (70–82)	80 ± 7 (72–87)	72 ± 14 (64–79)	78 ± 15 (67–82)	2.21, 0.15 0.06	0.47, 0.50 0.01	0.04, 0.83 0.00
Body height (m)	1.82 ± 0.04 (1.79–1.86)	1.83 ± 0.04 (1.80–1.86)	1.82 ± 0.06 (1.78–1.85)	1.82 ± 0.07 (1.78–1.85)	0.51, 0.48 0.02	0.06, 0.81 0.00	0.02, 0.89 0.00

(*/**/*** denotes *p* < 0.05/** *p* < 0.01/*** *p* < 0.001, respectively).

## Data Availability

The original contributions presented in this study are included in the Supplementary Table S1. Further inquiries can be directed to the corresponding author.

## Supplementary Materials

The following supporting information can be downloaded at: https://www.mdpi.com/article/10.3390/sports13060193/s1, Table S1.

## Abbreviations

The following abbreviations are used in this manuscript:

VO2max	Maximal oxygen uptake
EHF	European Handball Federation
GBPT	Game-based performance test
HRpeak	Peak heart rate
VO2peak	Peak oxygen uptake
IHF	International Handball Federation
